# Partner relationships, hopelessness, and health status strongly predict maternal well-being: an approach using light gradient boosting machine

**DOI:** 10.1038/s41598-023-44410-1

**Published:** 2023-10-09

**Authors:** Hikaru Ooba, Jota Maki, Takahiro Tabuchi, Hisashi Masuyama

**Affiliations:** 1 Department of Obstetrics and Gynecology, Okayama University Graduate School of Medicine, Dentistry and Pharmaceutical Sciences, Okayama, Japan; 2 Cancer Control Center, Osaka International Cancer Institute, Osaka, Osaka Japan

**Keywords:** Medical research, Risk factors, Health care, Quality of life

## Abstract

No recent study has explicitly focused on predicting the well-being of pregnant women. This study used data from an extensive online survey in Japan to examine the predictors of the subjective well-being of pregnant women. We developed and validated a light Gradient Boosting Machine (lightGBM) model using data from 400 pregnant women in 2020 to identify three factors that predict subjective well-being. The results confirmed that the model could predict subjective well-being in pregnant women with 84% accuracy. New variables that contributed significantly to this prediction were "partner help", "hopelessness," and "health status". A new lightGBM model was built with these three factors, trained and validated using data from 400 pregnant women in 2020, and predicted using data from 1791 pregnant women in 2021, with an accuracy of 88%. These factors were also significant risk factors for subjective well-being in the regression analysis adjusted for maternal age, region, parity, education level, and the presence of mental illness. Mediation analysis, with “hopelessness” as the mediator, showed that both “partner help” and “health status” directly and indirectly affected the outcome.

## Introduction

Significant changes have occurred since the emergence of the novel coronavirus disease (COVID-19). Existing literature provides mixed evidence on the impact of the pandemic on the mental well-being of the general population^1,2^. Specific demographics, such as young women, appear to be particularly susceptible to adverse mental health outcomes^1,3,4^. Additionally, pandemic-induced isolation and changes in working styles, including a rise in remote working, have altered stress responses and influenced family dynamics^5–7^.

Among these general trends, the well-being of pregnant women has emerged as a vital yet understudied area of concern. Pregnancy is a transitional period characterized by considerable physical and emotional changes. Even before the pandemic, these changes affected both maternal and infant health^8–11^. Recent studies have reported a decline in the subjective well-being of people affected by the COVID-19 pandemic^12^. Consequently, identifying the predictors of maternal well-being during these trying times has significant implications for public health policy making.

To address this, our study aimed to develop a machine learning model specifically designed to predict the subjective well-being of pregnant women during the COVID-19 pandemic. Machine learning offers a robust methodology for dissecting the complex interplay between the factors that affect well-being. Using this model, we also aimed to identify factors contributing to well-being, thereby informing early interventions to enhance the quality of life (QOL) of mothers and children, including unborn babies. This research builds on previous studies that found sociodemographic factors to be strongly associated with well-being in non-pregnant women^13^. Given the likelihood of shifting the determinants of well-being during the pandemic, our focus extends to the unique challenges faced by pregnant women.

## Methods

### Data source

We conducted a retrospective analysis of the Japan COVID-19 and Society Internet Survey (JACSIS), an annual survey initiated in 2020. The survey was managed by Rakuten Insight Corporation, a leading Internet research firm, and boasts a large, nationally representative sample pool of approximately 2.2 million panelists across various age groups, sexes, and socioeconomic statuses^14,15^. For the 2020 survey, we began collecting data on August 25, 2020. We randomly distributed surveys to a sample of 224,389 individuals, stratified by sex, age, and prefecture. The target number of respondents, stratified by sex, age, and prefecture, was set at 28,000, with an expected response rate of 12.5%, based on Japan's 2019 population distribution. This target was achieved on September 30, 2020. We filtered out invalid or inconsistent responses, such as male respondents claiming to be pregnant. Respondents identified as expectant mothers were further categorized based on their expected delivery dates. We then employed stratified random sampling to match Japan's national distribution, considering factors such as prefecture, sex, and age. The refined sample received the survey via email between October 15 and 25, 2020. The surveys were structured to require the completion of each question before progressing to the next question, eliminating the possibility of missing data due to non-response. To incentivize participation, respondents were offered credit points—referred to as “E-points”— that could be redeemed for online shopping or converted to cash. Another survey was conducted using a similar methodology from July 28 to August 30, 2021. We defined the target population based on data from two separate periods: Participants who answered as pregnant between October 15 and 25, 2020, were categorized as "2020 data," while those who answered as pregnant between July 28 and August 30, 2021, were categorized as "2021 data."

### Candidate determinants of well-being

We defined a binary outcome for well-being using a 10-point happiness scale. This scale was corroborated by a study that assessed happiness levels in a general Japanese sample using the JACSIS survey^16^. Based on this study, the median happiness score in the Japanese sample population was 7, with an interquartile range (IQR) of 6–8. Considering these findings, we defined a score of 7 or higher as good well-being (1) and a score of 6 or lower as poor well-being (0). To identify potential determinants of psychological well-being, we included a wide array of demographic, sociodemographic, and health-related variables such as age, sex, body mass index, marital status, educational level, occupation, and household income. We also integrated established mental health scales, such as the Kessler Psychological Distress Scale^17^ and the Edinburgh Postnatal Depression Scale^18^. Additionally, to capture the unique sociodemographic dynamics introduced by the COVID-19 pandemic, we considered factors such as anxiety regarding future household income and trust in both community and online interactions. The questionnaire comprised 552 items. Supplementary 1 provides the complete questionnaire.

### Statistical methods

Our analytical strategy comprised multiple steps, from preliminary descriptive statistics to advanced machine learning modeling.

### Descriptive analysis

Initially, we assessed the pregnant respondents' sociodemographic and health profiles in 2020 and 2021. We also examined the distribution of their subjective well-being scores during the COVID-19 pandemic.

### Model selection

We employed the Light Gradient Boosting Machine (LightGBM)^19^ to examine the prediction metrics of the outcome variable and to identify factors that contribute strongly to the prediction. Operating within a gradient-boosting framework, LightGBM uses an ensemble of decision trees to minimize a designated loss function. Its design optimization makes it highly efficient for computational processing and memory usage, making it ideal for large datasets. LightGBM can directly handle categorical variables, reducing the risk of overfitting in survey data rich in such elements. Furthermore, the gradient-boosting approach naturally captures the feature interactions. The model offers a wide range of hyperparameters, including solutions for class imbalances, thus facilitating task-specific performance optimization. Given these advantages, LightGBM emerged as the best-fitting model for our study. The decision tree algorithm calculates the probability that each sample belongs to a specific class. We classified samples into Class 1 if the calculated probability of belonging to Class 1 was 0.5 or higher, and into Class 0 if it was below 0.5.

### Selection of variables contributing to the prediction

Data from 2020 were randomly divided into training (64%), validation (16%), and test (20%) sets using the scikit-learn library's train_test_split function^20^. The model was trained using training and validation sets and evaluated on the test set. To counter overlearning, we applied Optuna^21^ for hyperparameter optimization. We used the SHapley Additive exPlanations (SHAP) package^22^ to quantify the importance of each variable. This method yielded insights into the influence of each variable on the model's predictions.

### Prediction of well-being

A new model based on the LightGBM was created, focusing exclusively on the three variables with the highest SHAP values as explanatory variables. The selection of these three variables was guided by their high SHAP values, which indicated a substantial influence on well-being. The model was trained using 80% of the 2020 data as the training set and the remaining 20% as the validation set. This trained model was employed to predict well-being levels in the 2021 data and assess its predictive accuracy. To examine the degree of influence of each predictor on well-being, a multivariate logistic regression model was constructed using the same three influential variables identified in the 2021 data, with well-being as the dependent variable. Odds ratios (ORs) for these variables were calculated to quantify their impact. The variance inflation factor (VIF)^23^ was calculated to address potential collinearity with a cutoff value of 10^24^.

Statistical significance was set at *P* < 0.05. All computations and visualizations were performed using Python version 3.8.16.

### Outcome

The primary outcome focused on the accuracy of the machine learning model, which was trained on the 2020 dataset and deployed to predict well-being in the 2021 dataset, using a binary well-being measure as the target variable. Secondary outcomes included precision, recall, F1 score, area under the receiver operating characteristic curve (ROC-AUC) for predictive accuracy, and odds ratios for key variables related to well-being. F1 score, a standard performance metric, represents the harmonic mean of precision and recall^25^.

1 $$accuracy = \frac{TP + TN}{{TP + FN + TN + FP}}$$

2 $$precision = \frac{TP}{{TP + FP}}$$

3 $$recall = \frac{TP}{{TP + FN}}$$

4 $$F_{1} \,score = 2 \cdot \frac{ Precision \cdot Recall }{{ Precision + Recall }}$$

TP, True Positive; FP, False Positive; TN, True Negative; FN, False Negative.

### Sensitivity analyses

We conducted a sensitivity analysis to validate the robustness and reliability of our machine learning model, particularly in the context of varying variables and thresholds. First, to investigate the impact of different model settings, we used various decision tree models to perform similar training and validation by comparing the metrics on the test data. We chose Random Forest^26^ and Extreme Gradient Boosting (XGBoost)^27^ as our models. Random Forest is a form of ensemble learning that trains multiple decision trees and integrates their results by averaging or taking a majority vote. XGBoost is a type of gradient boosting framework designed to train weak learners (usually decision trees) sequentially to correct errors from previous steps. Then we evaluated the robustness of our machine learning model by altering the cutoff thresholds for binary classification of the 10-point subjective well-being scale. Based on previous studies^16^, the thresholds were set to 6 and 8. We also tested the prediction accuracy of the model using the top two and four features.

We conducted a multivariate logistic regression analysis to control for possible confounders. These included education level^4^, gestational weeks^12^, parity^12^, maternal age^28^, mental illness^7^, and regional COVID-19 prevalence. The age threshold was set at ≥ 35 years, aligned with the common definition of geriatric pregnancy^29^. To perform a robust quantitative evaluation, we calculated doubly robust estimators (DRE)^30^ for the 10-point well-being scale. We transformed the key variables into a binary form as follows: "feeling hopeless" was scored as 1 for any response other than "not at all" when queried about hopelessness in the past 30 days; "lack of help from a partner" was scored as 1 for responses of "not at all" or "not very much" when questioned about partner support; "poor health status" was scored as 1 for descriptors of "not good" or "not too good" when asked about current health. We conducted a mediation analysis^31^ to quantify the direct and indirect effects of each variable on well-being, as there was a possibility that one variable could act as a mediator among the three key variables influencing the prediction. In the mediation analysis, we estimated two types of effects: direct effects, where the independent variable influences the dependent variable without the mediator, and indirect effects, where the influence occurs through the mediator. We also calculated bootstrap 95% confidence intervals (95% CI)^32^ with 1000 iterations for DRE, direct effects, and indirect effects.

### Ethical approval

All procedures were conducted in accordance with the ethical standards of the Declaration of Helsinki. The Osaka International Cancer Institute Research Ethics Committee reviewed and approved the study protocol (Approval No. 1412175183). All the participants provided written informed consent before responding to the online questionnaire. Furthermore, Internet survey agencies complied with the Act on the Protection of Personal Information in Japan^14^. We also ensured participant data anonymization and secure storage.

## Results

### Participants

**Figure 1:**
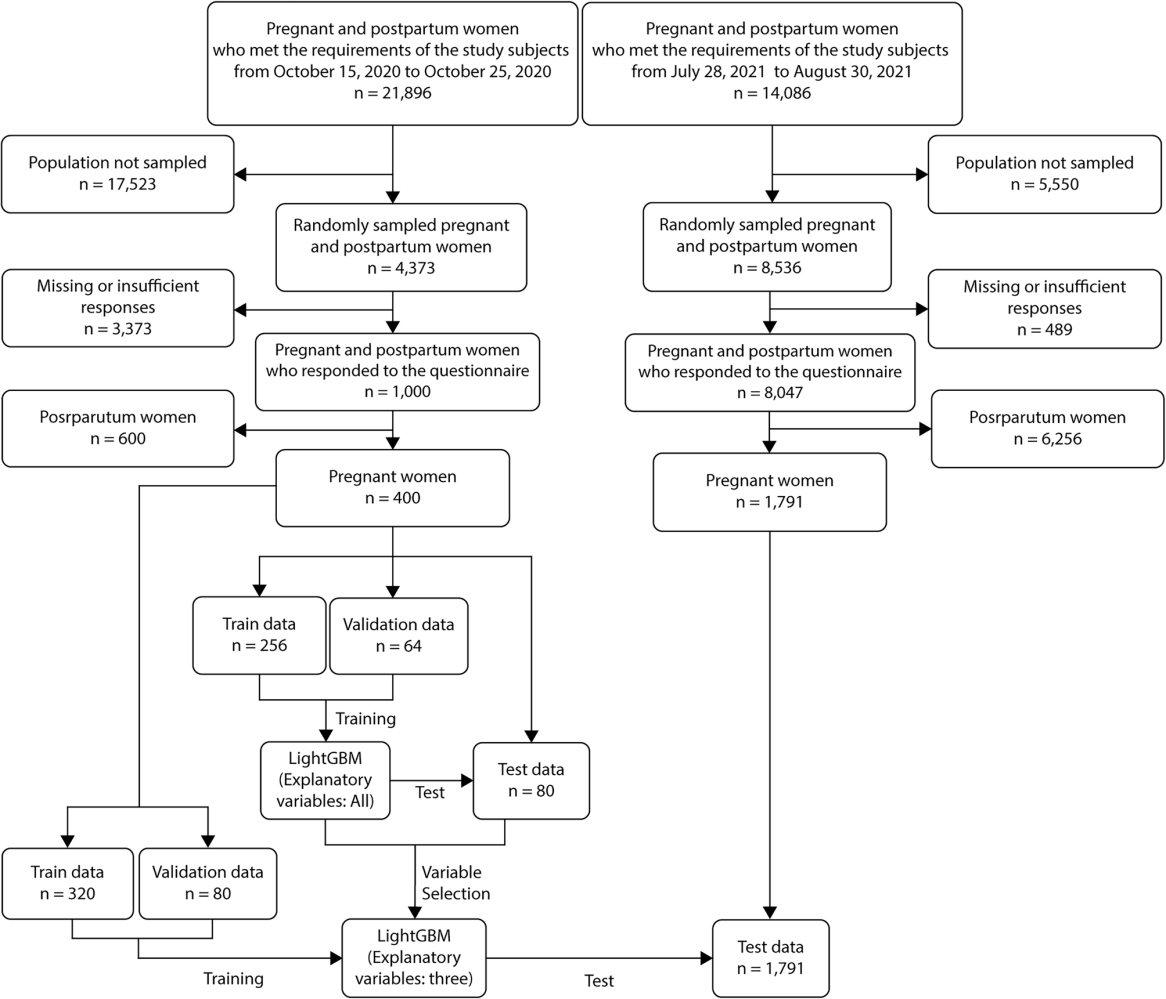
Inclusion of study participants.

In 2020, a panel of pregnant and parturient women were surveyed. We randomly sampled 4373 (20.0%) of 21,896 women who met the inclusion criteria. We excluded fraudulent and other responses and included 1000 (4.6%) participants in the final analysis, including 400 (1.8%) pregnant women. In 2021, of the 14,086 panelists who met the inclusion criteria, 8536 (60.6%) responded to the survey, and 8047 (57.1%) were selected for analysis after excluding fraudulent and other responses. Of these, 1791 (12.7%) were pregnant women (Fig. 1). The characteristics of the study population are summarized in Table 1.

**Table 1:** General characteristics of the study population (see Supplementary 2 for definitions of each region).

Characteristics	Pregnant women in 2020 (n = 400)	Pregnant women in 2021 (n = 1791)	*p*-value
Age, years (mean ± SD)	31.9 ± 4.9	31.5 ± 4.5	0.12
Gestation week (mean ± SD)	34.4 ± 5.1	33.3 ± 5.4	< 0.001
Parity			
Nullipara	199	942	0.33
Multipara	201	849	
Partner			
Present	397	1758	0.18
Absent	3	33	
Education			
With a college degree	191	962	0.04
Without a college degree	209	829	
Mental disorders			
Present	56	208	0.21
Absent	344	1583	
Region			
Hokkaido area	6	75	0.02
Tohoku area	26	93	
Kita-Kanto area	19	60	
Tokyo area	105	581	
Chubu-Hokuriku area	29	128	
Chukyo area	44	200	
Osaka area	71	283	
Keihan area	15	59	
Chugoku area	21	110	
Shikoku area	12	32	
Kyusyu-Okinawa area	52	170	

*SD* standard deviation.

### Prediction of well-being

**Figure 2:**
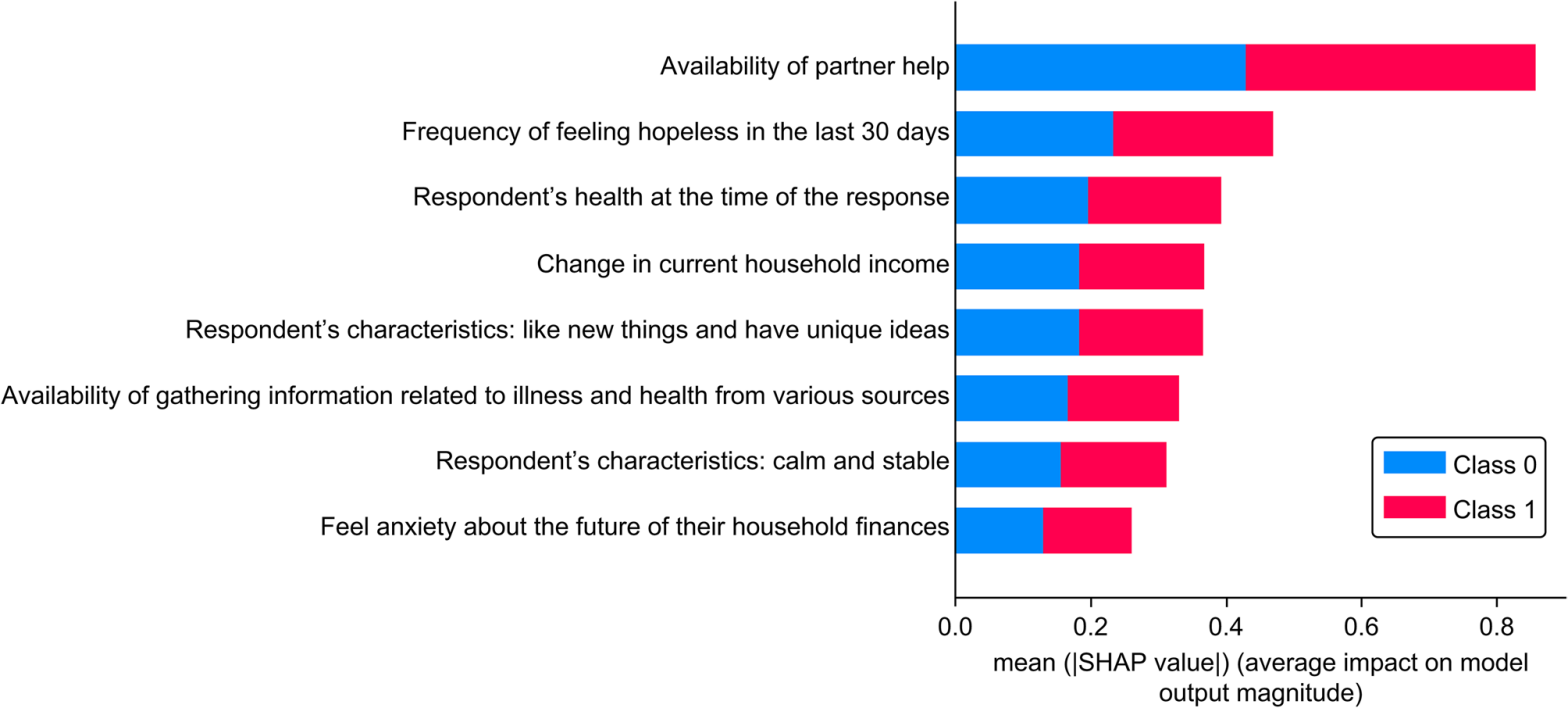
Order in which each variable contributes to the subjective well-being based on SHapley Additive exPlanations (SHAP) value. Red colors indicate positive values, while blue colors indicate negative values.

**Figure 3:**
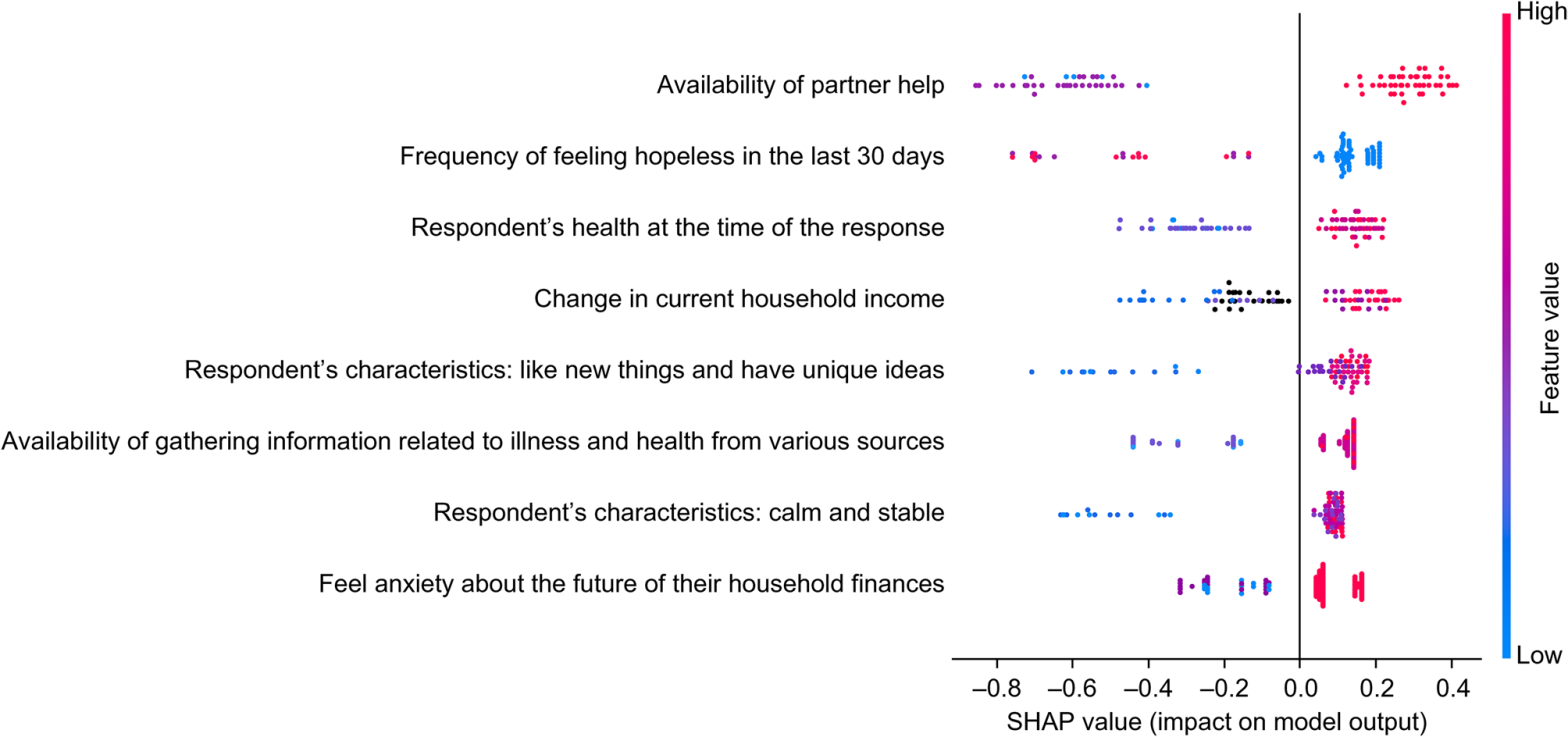
Violin plot of the SHapley Additive exPlanations (SHAP) value of each variable for predicting subjective well-being. The horizontal axis represents the impact on the objective variable model output, whereas the vertical axis shows a high contribution of the feature variables. Red colors indicate positive values, while blue colors indicate negative values. If the blue plots increase as the impact on machine learning output increases, this suggests that the objective and explanatory variables are negatively correlated.

**Figure 4:**
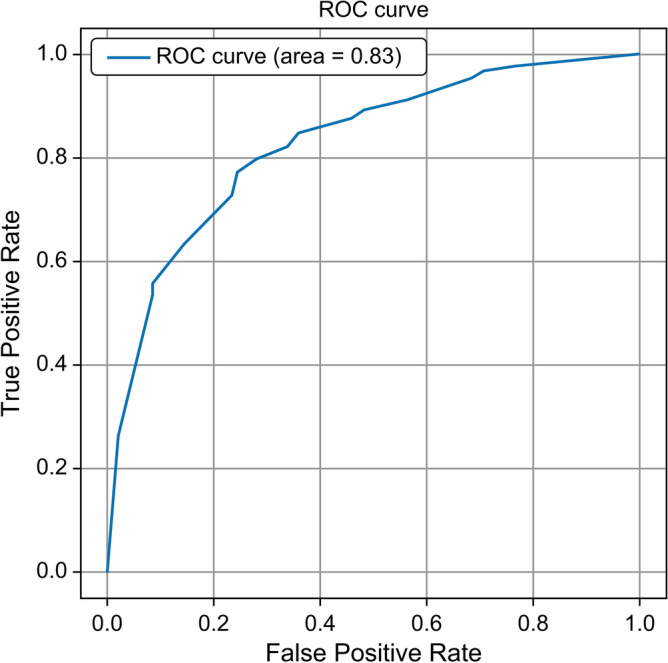
Receiver operating characteristic (ROC) curves for models trained using 2020 data and tested on 2021 data.

The distribution of the well-being is shown in Supplementary Fig. S1. The model demonstrated high accuracy indices when it was trained and validated using only 2020 data, with values of 0.84 for accuracy, 0.85 for precision, 0.97 for recall, 0.91 for F1 score, and 0.80 for ROC-AUC (Supplementary Fig. S2). Using SHAP values, we assessed the contributions of various characteristics to the objective variables (Figs. 2 and 3). We found that the characteristic with the most significant impact on the outcome variable was "availability of partner help." The next most significant characteristics were "frequency of feeling hopeless in the last 30 days" and "respondents’ health at the time of the response." We also collected information on COVID-19 status and vaccination within the questionnaire (Supplementary 1), but these did not contribute strongly to the prediction. Using these three variables, we built the LightGBM model again, trained and validated it with 2020 data, and predicted the 2021 data. The model also demonstrated high indices with values of 0.88 for accuracy, 0.92 for precision, 0.95 for recall, 0.93 for F1 score, and 0.83 for ROC-AUC (Table 2; Fig. 4). The results of the multivariate logistic regression analysis are presented in Table 3. We found that none of the multivariate logistic regression model variables had a VIF > 10.

**Table 2:** Prediction results for each parameter.

Model	Accuracy	Precision	Recall	F1-score	ROC-AUC
3 variables, threshold 7, LightGBM	0.88	0.92	0.95	0.93	0.83
3 variables, threshold 7, random forest	0.87	0.92	0.93	0.93	0.81
3 variables, threshold 7, XGBoost	0.87	0.92	0.94	0.93	0.81
2 variables, threshold 7, LightGBM	0.86	0.93	0.92	0.92	0.79
4 variables, threshold 7, LightGBM	0.89	0.92	0.96	0.94	0.84
3 variables, threshold 6, LightGBM	0.92	0.95	0.97	0.96	0.87
3 variables, threshold 8, LightGBM	0.78	0.85	0.86	0.85	0.79

For all patterns, the machine learning models were trained and validated using 2020 data and tested on 2021 data.

*ROC-AUC* area under the receiver operating characteristic curve, *LightGBM* Light Gradient Boosting Machine, *XGBoost* Extreme Gradient Boosting.

**Table 3:** Results of the multivariate logistic regression analysis.

Factor	Coefficient	*p*-value	95% CI	VIF
Intercept	0.018	< 0.001	0.001–0.034	
Frequency of feeling hopeless	1.747	< 0.001	1.487–2.052	4.390
Availability of partner help	2.811	< 0.001	2.259–3.497	4.851
Respondent's health	1.963	< 0.001	1.627–2.368	5.303

*CI* confidence interval, *VIF* variance inflation factor.

### Sensitivity analyses

Table 2 shows the prediction accuracy for the 2021 data when the models, well-being thresholds, and number of features were varied. Table 4 shows the results of the multivariate logistic regression analysis when covariates were added as explanatory variables. As the VIF was > 10, we did not include “gestation weeks” in the analysis. “Parity,” “education level,” and “mental disorders” were significantly different, but these did not affect the trends in odds ratios for the three variables. The DREs were − 1.65 (95% CI − 2.22, − 1.12) for "lack of help from a partner," − 1.32 (95% CI − 1.60, − 1.06) for "feeling hopeless," and − 0.73 (95% CI − 1.03, − 0.42) for "poor health status," respectively. In the mediation analysis, the direct effect of “lack of help from a partner” on “Well-being” was − 1.50 (95% CI − 2.12, − 1.21), and the indirect effect through “feeling hopeless” was − 0.40 (95% CI − 0.60, − 0.27). For “poor health status” as the explanatory variable, the direct effect was − 0.97 (95% CI − 1.43, − 0.73), and the indirect effect was − 0.59 (95% CI − 0.63, − 0.35).

**Table 4:** Results of the multivariate logistic regression analysis when age, region, parity, education level, and presence of depression are included.

Factor	Coefficient	*p*-value	95% CI	VIF
Intercept	0.002	< 0.001	0.001–0.005	
Frequency of feeling hopeless	1.717	< 0.001	1.454–2.028	4.581
Availability of partner help	2.902	< 0.001	2.312–3.643	5.668
Respondent's health	1.933	< 0.001	1.597–2.340	6.230
Age ≥ 35 years	1.074	0.730	0.716–1.611	1.364
Region	1.036	0.314	0.968–1.110	3.985
Parity	0.634	0.015	0.439–0.917	1.973
Education level	0.587	0.003	0.410–0.839	1.191
Mental disorders	1.951	0.004	1.253–3.025	1.197

*CI* confidence interval, *VIF* variance inflation factor.

## Discussion

This study is novel because it shows that a machine learning model trained using data collected from pregnant women can predict heterochronous well-being with 88% accuracy. The variables that significantly contributed to this prediction were “lack of help from a partner,” “feeling hopeless,” and “poor health.” Multivariate logistic regression analysis using these variables as explanatory variables also confirmed that they were significantly associated with subjective well-being. These trends remained consistent after adjusting for age, region, parity, educational level, and history of mental illness. Furthermore, DRE showed a trend toward lower well-being when any prediction variable was negatively skewed. Mediation analysis, assuming 'feeling hopeless' as the mediator, indicated a trend toward lower well-being when the predictors were negatively skewed. Additionally, the results showed that each variable exerted both direct and indirect effects on maternal well-being through 'feeling hopeless,' with the direct effects being more substantial than the indirect effects. This trend is consistent with the results of the summary plots of the SHAP values from the machine learning model.

These results suggest that during the COVID-19 pandemic, pregnant women's family relationships, mental state, and health may be strong predictors of subjective well-being. Previous online survey using the WHO-5 Well-Being Index and the Cambridge Worry Scale^33^ noted a high percentage of pregnant women experiencing low well-being during the COVID-19 pandemic. A study conducted during the COVID-19 pandemic that investigated happiness among people without limiting the participants to pregnant women^16^ suggested that social factors, like the presence of a partner or trust in the community, are positive determinants of happiness during the pandemic. The authors pointed out that in many regions, physical isolation measures to contain the spread of COVID-19 led to a reduction in social interactions and an increase in the possibility of psychological isolation. A study analyzing emotions in posts on online support forums for pregnant women during the COVID-19 pandemic^34^ noted an abundance of negative sentiments. These stemmed from distress related to the despair due to the loss of social and familial support, and anticipated grief from family and support structure changes. Due to their higher risk of severe complications from COVID-19 infection, perinatal women were particularly prone to being physically distanced both for their own safety and that of their children. As these findings indicate, the presence of a partner strongly influenced the well-being of pregnant women, and our results emphasized this aspect. A sense of hopelessness may also influence the decline of well-being due to decreased social interaction and increased psychological isolation. A meta-analysis^35^ of pregnant women's QOL conducted before the COVID-19 pandemic found that partner support^36^ was a factor that improved QOL, and physical factors (e.g., complications during pregnancy, physical symptoms such as nausea and vomiting^37^, and sleep disturbances^38^) and psychological factors (e.g., anxiety, stress, and depression during pregnancy^7^) were associated with reduced QOL. Another research^5^ suggests that during an infectious disease epidemic, pregnant women may be particularly distressed because of concerns about their health. Social capital has been suggested to positively impact health status because of its knowledge transfer channels, reinforced behavioral norms, and community cohesion^39^. Recent studies^40^ have indicated that community cohesion is linked to the increased use of preventive health care. In terms of health status, social capital may also have an impact^16^.

Our study had several limitations. First, this was an exploratory study using machine learning methods to examine factors affecting pregnant women’s subjective well-being during the COVID-19 pandemic, and not a study to evaluate causal relationships. Therefore, it is unclear whether improving the predictors identified in this study would improve pregnant women’s well-being. Second, this was a cross-sectional study based on web-based self-reports, and the small sample size may involved selection bias. Although stratified random sampling was conducted, the 2020 survey yielded only 400 pregnant women due to budget constraints. The 2021 survey enabled us to collect more data. Despite the different periods and numbers of people in the data used for learning and validation and the data used for testing, predictions can be made with high accuracy, and we believe that qualitative trends are captured. Third, it is essential to note that well-being is a multifaceted concept, and overall subjective well-being, as rated on a 10-point Likert scale, does not capture all aspects of pregnant women's well-being. However, social desirability bias can be reduced by using anonymous questionnaires on a simple scale^41^. Fourth, 2021 test data may include data from pregnant women in 2020. However, since it usually takes 10 months from pregnancy to delivery, and it is rare for a woman to have another pregnancy immediately after childbirth, we believe that even if the test data contain duplicates, the number is likely to be small. Fifth, because our study retrospectively analyzed data from the JACSIS, our findings may not be generalizable to non-Japanese populations.

## Conclusion

We developed a highly accurate model to predict the subjective well-being of pregnant women. Partner’s help, pregnant women's sense of hopelessness, and pregnant women's health status significantly contributed to this prediction.

## Supplementary Information

Supplementary Information.

## Data Availability

The datasets generated and/or analyzed during the current study are available from the corresponding author upon reasonable request.
