# Author Correction: Partner relationships, hopelessness, and health status strongly predict maternal well-being: an approach using light gradient boosting machine

**DOI:** 10.1038/s41598-025-93454-y

**Published:** 2025-03-13

**Authors:** Hikaru Ooba, Jota Maki, Takahiro Tabuchi, Hisashi Masuyama

**Affiliations:** 1 Department of Obstetrics and Gynecology, Graduate School of Medicine, Dentistry and Pharmaceutical Sciences, Okayama University , Okayama, Japan; 2 Cancer Control Center, Osaka International Cancer Institute, Osaka, Osaka Japan

Correction to: *Scientific Reports* 10.1038/s41598-023-44410-1, published online 09 October 2023

The original version of this Article contained an error in Affiliation 1, which was incorrectly given as “Department of Obstetrics and Gynecology, Okayama University Hospital, Okayama, Okayama, Japan.”. The correct affiliation is listed below:

“Department of Obstetrics and Gynecology, Graduate School of Medicine, Dentistry and Pharmaceutical Sciences, Okayama University, Okayama, Japan.”

The original Article has been corrected.

