# Supplementary material for: Partner relationships, hopelessness, and health status strongly predict maternal well-being: an approach using light gradient boosting machine
Source: Sci Rep. 2023 Oct 9;13:17032. doi: 10.1038/s41598-023-44410-1 (PMC10562477; doi:10.1038/s41598-023-44410-1)

Partner Relationships, Hopelessness, and Health Status Strongly Predict Maternal Well-Being: Approach using Light Gradient Boosting Machine.

Hikaru Ooba, MD[1], Jota Maki, MD, PhD[1,*], Takahiro Tabuchi, MD, PhD[2], Hisashi Masuyama, MD, PhD[1]

[1] Department of Obstetrics and Gynecology, Okayama University Hospital, Okayama, Japan

[2]Cancer Control Center, Osaka International Cancer Institute, Osaka, Japan

[*]**Corresponding Author:** Jota Maki, MD, PhD

Assistant Professor, Department of Obstetrics and Gynecology, Okayama University Hospital

2-5-1 Shikata-cho, Kita-Ku, 700-8558, Okayama City, Okayama Prefecture, Japan

Email: jotamaki@okayama-u.ac.jp

Supplemental contents:

Supplementary notes:

Supplementary 1: Survey questionnaires used in this study.

Supplementary 2: The definition of each region.

Supplementary figures:

Supplementary figure S1: Distribution of subjective well-being of pregnant women in 2020 and 2021

Supplementary figure S2: Receiver operating characteristic (ROC) curves for models trained and tested using 2020 data

Supplementary 1: Survey questionnaires
(Questions about post-partum were excluded.)

SC1. (Screening) Please describe your current situation
*If you gave birth after October 2019, are currently pregnant, and expect to give birth by March 2021, please give priority to [I gave birth] and answer the question.
<Options>
[gave birth]
1. You gave birth between October 2019 and March 2020.
2. You gave birth between April 2020 and May 2020.
3. You gave birth on or after June 2020.
[Pregnant]
4. You expected to give birth by the end of March 2021
[Other]
5. Not applicable to the above

Please answer today's date (Date you answered) [ ] Year [ ] Month [ ] Day
(Rakuten Insight automatically collects information on the date and time of year)

(Q1-1) When is (was) the due date of your childbirth? [ ] year [ ] month [ ] day

(Q2) How many people, including yourself, do you usually live with and make a living with?
1. Spouse (including common-law relationship)
2. Child (under elementary school age)
3. Child (elementary school age and older)
4. Parents/Parents-in-law
5. Grandchild
6. Grandparents/Grandparents-in-law
7. Siblings
8. Others (friends, etc.)

(Q4) How old are each of your children who live with you?
*Please answer in order from the youngest child.
Child 1: ____ years old
Child 2: ____ years old
Child 3: ____ years old
Child 4: ____ years old
Child 5: ____ years old

(Q5) Did you work during the following periods?

1. January 2019 to December 2019
2. January to March 2020
3. April to May 2020
4. June to August 2020
5. September 2020 to present

<Choice>

1. Yes
2. No

Question display condition: 1-5 of (Q5) is 1.

(Q5-1) Choose one that applies to your work situation (including work on leave) from January 2019 to the present. If you have two or more jobs, please answer about one main job. If you are a student and have a job, please answer about whichever one you spend the most time at. If you have left a job since January 2019 due to pregnancy or childbirth or are on a leave of absence, please answer about your work situation up until immediately before.

1. Directors of companies and other organizations (excluding self-employed persons)
2. Self-employed
3. Assistance with own business
4. Full-time employees and other regular staff (management)
5. Regular employees and other regular staff (other than management)
6. Temporary workers at worker dispatch offices
7. Contract employees and contract employees
8. Part-time job
9. Wage work at home (inside job)
10. Students (including students who failed the entrance exam)

Question display condition: 1-5 of (Q5) is 1.

(Q6-1) Tell us about your current main type of work. If you have two or more jobs, please answer about one main job. *If you have resigned or are on leave due to this pregnancy or childbirth, please answer about your work situation up until immediately before.

1. Public servant
2. Agriculture, forestry, fisheries, and fishing
3. Mining industry
4. Construction industry
5. Manufacturing industry
6. Electricity, gas, heat supply, and water supply
7. Telecommunications industry
8. Transportation business

9. Wholesale business
10. Retail trade
11. Finance business
12. Insurance business
13. Real estate business
14. Restaurant business (with alcoholic beverages served)
15. Restaurant business (no alcohol served)
16. Lodging business
17. Medical care
18. Welfare
19. Education, learning support industry
20. Other services (not elsewhere classified)

(Q6-2) Which of the following applies to the size of your place of business where you work? *If you resigned or are on leave due to this pregnancy or childbirth, please answer about your work situation up until immediately before.

1. 1 person
2. 2-4 persons
3. 5-29 persons
4. 30-49 persons
5. 50-99 persons
6. 100 to 299 persons
7. 300-499 persons
8. 500 to 999 persons
9. More than 1,000 persons

(Q7) Tell us your zip code.

Question display condition: 1-5 of (Q5) is 1.

(Q7-1) Tell us the zip code of your place of work. *If you have resigned or are on leave due to this pregnancy or childbirth, please answer about your work situation up to immediately before.

Question display condition: 1-5 of (Q5) is 1.

(Q8) Choose the one that most closely describes your job. *If you resigned or are on a leave of absence due to this pregnancy or childbirth, please answer about your work situation up to immediately before.

1. Mainly desk work (clerical and computer work)
2. Work that mainly involves talking to people (sales and marketing)
3. Mainly physically demanding work (e.g., working in production, nursing care, etc.)

(Q9) About your job (including part-time work).

Tell us your average hours worked per week for each of the following. For example, 5 hours per day x 5 days/week = 25 hours per week

1. What were the average total hours working per week before December 2019 (Question display condition: 1 in (Q5) is 1)
2. What were the average total hours working per week in April-May 2020 (Question display condition: 3 in (Q5) is 1)
3. What were the average total hours working per week in the last month (Question display condition: (Q5) 5 is 1)
4. What were your desired total actual hours working per week (Question display condition: any 1-5 of (Q5) is 1)

<Choice>

1. 0 hours per week (no actual working hours)
2. Less than 20 hours per week
3. 20-24 hours per week
4. 25 to 29 hours per week
5. 30-34 hours per week
6. 35-39 hours per week
7. 40-44 hours per week
8. 45-49 hours per week
9. 50-59 hours per week
10. 60-69 hours per week
11. 70 hours per week or more

Question display condition: 5 in (Q5) is 1.

(Q11) Tell us about your work situation and satisfaction.

*If you have resigned or are on leave due to this pregnancy or childbirth, please answer the following questions regarding your work situation up to the time immediately before.

1. Very much work to be done.
2. Work cannot be processed in time.
3. Heavy workload associated with late night hours (10 pm - 5 am).
4. Hard work.
5. Work requires attention focus.
6. Very physical job.
7. Work at own pace.
8. Able to decide the order and method of work by yourself.
9. Able to reflect on your own opinions on workplace work policies.
10. In the workplace, there is a willingness to work together
11. In the workplace, you understand and recognize each other.

12. The workplace can share work-related information.
13. The work environment (noise, lighting, humidity, ventilation, etc.) is not good
14. I feel energized and energized at work
15. I feel proud of my work
16. I am bullied at work (including sexual harassment and power harassment)
17. Someone is being bullied at work (including sexual harassment and power harassment)
18. I fear losing my job.
19. I am happy with my job.
20. I am happy with my home life.

<Choice>

1. That is correct.
2. Maybe.
3. My situation is somewhat different
4. Not correct.

Question display condition: 1-5 of (Q5) is 1.

(Q12) Please select the option that applies to each of the following.

For those who resigned or are on leave due to pregnancy or childbirth, please answer about your work situation up to the time immediately before.

1. How comfortable are you talking to your supervisor?
2. How comfortable are you talking with your co-workers?
3. How comfortable are you talking with your spouse, family, or friends?
4. How reliable is your supervisor when you are in trouble?
5. How dependable are your co-workers when you are in trouble?
6. How dependable are your spouse, family, and friends when you are in trouble?
7. How willing is your boss to listen to you when you discuss your personal problems with them?
8. How willing are your co-workers to listen to you when you discuss your personal problems with them?
9. How willing are your spouse, family, and friends to listen to you when you discuss your personal problems?

<Choice>

1. Very
2. Considerably
3. More or less
4. Not at all

(Q13) Do you currently have a spouse (husband or wife)? Spouse includes those living together as husband and wife but have not yet registered their marriage.

<Choice>

1. I have a spouse.
2. Unmarried
3. Bereavement (bereavement occurred before March 2020)
4. Bereavement (bereavement occurred after April 2020)
5. Divorced (divorced before March 2020)
6. Divorced (divorced after April 2020)

(Q14) Check your medical insurance coverage with your insurance card or union membership card. Answer only one question that applies.

1. National Health Insurance (Municipal)
2. National Health Insurance (Union)
3. Employee insurance (National Health Insurance Association)
4. Employee insurance (health insurance association)
5. Employee insurance (Mutual aid association)
6. Employee insurance (seamen's insurance, etc.)
7. Public assistance
8. Uninsured (no medical insurance, medical insurance still expired)
9. Other

(Q15) Tell us about the last school you graduated (or dropped out of) or are currently attending.

1. Junior high school
2. Private high school
3. National and public high schools
4. Vocational school
5. College / College of technology
6. Private university
7. National university
8. Public universities (prefectural, municipal, etc.)
9. Graduate school
10. Other

(Q16) Regarding the schools you answered above, please choose the one that applies to you from the following.

1. Graduation/Completion
2. Leaving school during a term
3. While in school (including leave of absence, etc.)

(Q17) Choose the second from the bottom from the following options.

1. A
2. B
3. C
4. D
5. E

(Q18) Please select one item that applies to your current residence.
1. Owner-occupied house (single-family home)
2. Owner-occupied house (condominium)
3. Rental housing (private apartments and condominiums)
4. Rental housing (public housing complexes and apartments)
5. Lodging and renting a room
6. Company housing, dormitories, government housing, official housing
7. Other

(Q19) How many rooms (in total) are in your house? (Toilets, bathrooms, and kitchens are not included in the number of rooms. For example, the number of rooms in a 2LDK house is 2)

(　　　)Rooms

(Q20) Please describe your current work situation.

1. Mainly working at the workplace
2. Working mainly from home
3. On maternity or childcare leave
4. On vacation or at home
5. Had been working before pregnancy but stopped working after pregnancy and childbirth
6. Not working, even before pregnancy

Question display condition: 1-5, 12-18: Gender is female.

Question display condition: 6-11: (Q20) is one of 1-5

(Q21) (Pregnancy Care and Support) Did you experience any of the following from this pregnancy to delivery?

1. This pregnancy was a wanted or planned pregnancy.
2. This pregnancy was the result of fertility treatment.
3. You reduced the number of antenatal checkups.
4. Failed to attend or reduce motherhood or parenthood classes during the pregnancy.
5. You felt you had no choice but to follow the doctor's instructions about delivering the baby and breastfeeding while in the hospital without telling them what you wanted.
6. During pregnancy, you offered to change your working style (staggered commute, shorter hours, telecommuting, staying at home, taking leave, etc.) to your employer.
7. During your pregnancy, your workplace changed your working style (staggered commute, shorter hours, telecommuting, staying at home, taking leave, etc.) regardless of your wishes.
8. Harassment, such as sarcasm, due to pregnancy or childbirth or when using the maternity/paternity leave system
9. When you requested pregnancy, maternity, or childcare leave or requested a change in work style, you were treated contrary to your wishes, such as being demoted or ordered to stay at home.

10. Your attending physician wrote the maternal Health Care Guidance Items Communication Card to the workplace.
11. You were concerned that you or your baby could get a new type of coronavirus infection at work or on the commute to work.
12. You were concerned about the possibility of a new type of coronavirus infection in you or your baby through your family.
13. Your partner or husband telecommutes or teleworks.
14. You wanted to talk to a friend or acquaintance about your pregnancy problems but could not.
15. You were scheduled to give birth at the medical institution where you had received your antenatal checkup but were transferred to a different hospital when it stopped accepting deliveries.
16. Gave up or refrained from having a homebirth.
17. The medical institution where you gave birth at your homeland refused to accept you.
18. You wished to have the birth with your husband, or you wished to do so.

<Choice>

1. Yes
2. No

(Q23) Did you receive any of the following diagnoses or indications from your doctor during this pregnancy? *Please answer this question while referring to your Maternal and Child Health Handbook.

1. Multiple pregnancies of twins or triplets or more
2. Aggravation of a pre-pregnancy illness
3. Morning sickness requiring hospitalization
4. Anemia requiring hospitalization
5. Pregnancy-induced hypertension
6. Gestational diabetes mellitus
7. Impending miscarriage (almost miscarriage at less than 22 weeks gestation)
8. Impending premature labor (almost going into premature labor)
9. Placenta previa
10. Premature separation of the prevailing placenta
11. Water breaking during pregnancy
12. Pre-eclampsia
13. Health problems other than those listed above that required hospitalization
14. Health problems of the baby in the tummy

<Choice>

1. Yes
2. No

(Q25) How do you feel about breastfeeding? *If you have already given birth, please answer how you felt about it when you were pregnant.

1. Would very much like to breastfeed (or thought to)

2. Want to breastfeed if breast milk is produced (or thought to)
3. Want baby to be raised on formula (or thought to)
4. Want baby (or thought to want) to be raised on both breast milk and formula (or thought to)
5. Not particularly thought about (did not think about)

(Q30) For the following questions, please think of your family and choose the appropriate options. Family members can include family members who live with you and family members who live separately.

1. If you need help, can your family help you?
2. Are you happy to discuss and share hardships with your family?
3. Does your family help you when you try something new?
4. Is your family responsive to your emotions (e.g., anger, loneliness, love, etc.)?
5. Is there time for a family reunion?
6. Does your partner (husband or de facto partner) help you in your need
7. Does your partner respond to your feelings (happiness, joy, sadness, anger, etc.)?
8. Does your partner perform household chores, child care, and other domestic roles?

<Choice>

1. Yes, always.
2. Yes, sometimes.
3. No, not really.
4. No, not at all.

(Q31) Have you had any following symptoms within the last month?

1. High fever
2. Physical lethargy and malaise
3. Sore throat
4. Cough
5. Breathlessness
6. Nausea/Vomiting
7. Toothache
8. Abdominal pain, diarrhea
9. Taste and smell disorders

<Choice>

1. Yes
2. No

(Q32) Were there any medical or illness-related events that you could not do or you postponed during the April-May 2020 period?

1. I ran out of my regular medication.
2. My chronic illness worsened.
3. Could not go (refrained from going) to the hospital as scheduled

4. Unexpected symptoms or medical conditions that prevented (refrained from) going to the hospital or receiving a doctor's visit
5. Hospitalization was not possible (was postponed)
6. Treatment (surgery) could not be performed (postponed)
7. Treatment (other than surgery) could not be performed (postponed)
8. Could not (or refrained from) going to the dentist's office

<Options>

1. Yes
2. No

Question display condition: 1, 4, or 5 in (Q5) is 1

(Q33) Have you experienced any of the following events since April 2020 compared to before?

1. Increased physical strain of work
2. Decreased physical strain of work
3. Increased emotional burden of work
4. Decreased emotional burden of work
5. Worked at home (at home) before March 2020
6. Started working at home (from home) after April 2020
7. Increased telecommuting (working at home)
8. Telework (remote work) other than telecommuting before March 2020.
9. Started teleworking (remote work) other than telecommuting before March 2020
10. Increased telework (remote work) other than telecommuting
11. Telecommuting, staggered commutes, etc., to reduce commuting burdens.
12. An online meeting about the job.
13. Had to go to work for stamps and other paperwork.
14. Had to go to work to use the system.
15. Due to the new coronavirus problem, the number of scheduled jobs decreased (or disappeared).
16. Due to the new coronavirus problem, you took leave or furlough work.
17. Due to the new coronavirus problem, you resigned from your work (including contract not renewed, etc.).
18. Due to the new coronavirus problem, you changed jobs (including not renewing contracts, etc.).

<Choice>

1. Yes
2. No

Question display condition: 8 is displayed when the sum of 2, 3, and 5 in (Q3) is greater than 1.

Question display condition: Display 1-7 with male and female.

(Q34) From April to May 2020 and from June to the present, how much time did you spend (on average) per day doing the following? *VDT work refers to using information devices such as PCs, smartphones,

and tablets for data entry, searching, etc. creating and editing text, and images, etc. programming and monitoring, playing games, and using social networking service (SNS).

<Period of Emergency Declaration (around April-May 2020)>

1. VDT at work or study
2. VDT outside of work or study
3. Sitting time
4. Time spent walking or standing
5. Time for physical labor or strenuous sports
6. Times for sleep
7. Times for housework: mainly household chores (cooking, laundry, cleaning, etc.)
8. Times for childcare: mainly raising children (e.g., taking care of children and grandchildren, playing with them, etc.)

<Choice>

1. None (0 hours)
2. Less than 30 minutes per day
3. About 30 minutes per day
4. 1 hour per day
5. 2 hours per day
6. 3 hours per day
7. 4-5 hours per day
8. 6-7 hours per day
9. 8-9 hours per day
10. 10-11 hours per day
11. At least 12 hours per day
12. I don't know.

<June 2020 – present>

1. VDT at work or study
2. VDT outside of work or study
3. Sitting time
4. Time spent walking or standing
5. Time for physical labor or strenuous sports
6. Time for sleep
7. Time for housework: mainly household chores (cooking, laundry, cleaning, etc.)
8. Time for childcare: mainly raising children (e.g., taking care of children and grandchildren, playing with them, etc.)

<Choice>

1. None (0 hours)
2. Less than 30 minutes per day
3. About 30 minutes per day

4. 1 hour per day
5. 2 hours per day
6. 3 hours per day
7. 4-5 hours per day
8. 6-7 hours per day
9. 8-9 hours per day
10. 10-11 hours per day
11. At least 12 hours per day
12. I don't know.

(Q35) Have you taken any of the following actions in the last month?

1. Disinfected hands and fingers with rubbing alcohol
2. Hand washing with soap/hand soap for more than 15 seconds
3. Gargled when returning home, etc.
4. Cough etiquette performed.
5. Avoided touching eyes, nose, and mouth with unwashed hands
6. Disinfected doorknobs and other items easily touched by human hands.
7. Opened the windows to ventilate the room.
8. Wore a mask in the presence of people.
9. Planned an upcoming trip.
10. Refrained from unnecessary outings and business trips
11. Avoided talking or vocalizing at close range (within 1 meter)
12. Tried to socially distance (at least 2 meters away from people)
13. Avoided meeting with people considered to be at high risk of infection
14. Tried not to go to crowded places.
15. Ate a nutritionally balanced diet.
16. Tried to lead a regular life.
17. Had breakfast each day.
18. Refrained from using a restaurant's interior
19. Avoided cigarette smoke (passive smoking)

<Choice>

1. Always
2. Occasionally
3. Rarely.
4. I didn't do it at all

Question display condition: Gender is male or female, and 1 to 9 are displayed.

Question display condition: 8 is displayed when 2+3 in (Q3) is greater than 1.

Question display condition: 1 in (Q3) is 1 or more, and 10 is displayed.

(Q36) How has the following changed in the last month compared to before January 2020?

1. Opportunities to exercise (physical activity)
2. Hours of sleep
3. Inability to sleep well
4. Not eating breakfast.
5. Amount and frequency of drinking
6. Amount and frequency of smoking
7. Spending time with family
8. Time spent with children
9. Stress about being with family.
10. Number of marital quarrels

<Choice>

1. Increased
2. Same as before
3. Decreased

(Q37) If your household income in 2019 is 100, how has your current household income changed? For example, if it decreased by half, please answer 50; if it doubled, please answer 200. (Please use half-width numbers)

(   )

Options: I don't know.

(Q38) Did you receive a seasonal flu shot last winter or this spring (between October 2019 and March 2020)?

<Choice>

1. Received
2. Not received
3. I don't know

(Q39) We would like to ask those who answered that their child is "0-14 years old".
Did your children receive a seasonal flu shot between last winter and this spring (between October 2019 and March 2020)?

<Choice>

1. Received
2. Not received.
3. I don't know.

Question display condition: 2+3 in (Q3) is greater than 1

(Q40) Has the crisis caused by the new coronavirus infection in your area made a difference in your child's life?

<Choice>

1. There was no change.

2. There was a rather positive change (Specify:)
3. There was a rather negative change (Specify:)
4. I don't know.

(Q41) We would like to ask those who answered that their child is "0-14 years old".

Tell us about your child's school and daily life situation during the emergency declaration period (around April to May 2020). *If more than one child applies, please answer below for the child with the highest priority. Highest priority: elementary school students (upper grades) / Second priority and subsequent order: elementary school students (lower rates) - junior high school students - high school students - kindergarten and nursery school students - college students - others

1. Entrance ceremony was canceled (you could not attend)
2. Schools, kindergartens, and nursery schools were closed
3. Refrained from going to school, kindergarten, or preschool
4. Children left at grandparents' or relatives' homes due to school closure or school holidays
5. Children were left in the care of babysitters, family support, school-age children, or other childcare providers due to school closures or vacations.
6. I could not afford to care for my children's learning at home.
7. Children were receiving some online classes or other educational content
8. Children spend a lot of time on TV and playing video games.
9. There were spaces (rooms, etc.) where children could study and spend time.
10. Children were taking cram school classes.
11. Children were taking English classes, abacus, and other lessons.
12. There were times when the child was injured.
13. There were times when I couldn't get my children to eat their food.
14. Child's mental health became unstable
15. The child was violent.
16. The child spoke out of turn.
17. The child was subjected to violence.
18. The child was verbally abused.
19. Child's motivation to learn has decreased
20. Child stopped going to school (stopped attending school)

<Choice>

1. Yes
2. No
3. I don't know.

Question display condition: 1-5 in (Q4) is less than 18

(Q42) Does your family do the following things to your children?

1. Slapping the body (buttocks, hands, head, face, etc.)
2. Give a loud scolding

3. Strike
4. Lock outside
5. Ignore.
6. Not feeding them.
7. Repeatedly say things that are hurtful to the child
8. Leaving children alone at night
9. Have a big fight in front of the children
10. Smoking cigarettes in front of children

<Choice>

1. Often.
2. Occasionally.
3. Rarely.
4. Not at all.

Question display condition: Gender is male or female, and the display is for all except 3 and 13.

Question display condition: 3 is displayed when (Q5-1) is 10 or (Q3) 2 + (Q3) 3 is 1 or more.

Question display condition: 2 in (Q3) + 3 in (Q3) + 5 in (Q3) is greater than 1, and 13 is displayed.

(Q43) Did you experience any of the following events between April 2020 and the present?

1. Unpaid salaries occurred.
2. Didn’t or don't have enough money to buy the necessities of life.
3. Can no longer pay my tuition.
4. Can no longer pay my rent/mortgage.
5. Don't have enough money for medical care.
6. Don't have enough money for dental care.
7. Received online medical care.
8. Participated in an online drinking party.
9. You or your family member or colleague have been infected with a novel coronavirus
10. Used or using COCOA, an app for tracking new coronavirus infections.
11. Didn’t or don’t have enough money for food.
12. Felt too much of a burden on the household.
13. Felt that the burden of childcare (care and involvement of children and grandchildren) was too much
14. Felt anxious about the future of our household finances
15. There were times when you wanted to die.

<Choice>

1. I experienced it for the first time.
2. I experienced it for a while.
3. I did not experience it.

(Q44) Do any of the following apply to you?

1. You are terrified of the new coronavirus.

2. The new coronavirus makes you uncomfortable.
3. Your hands sweat when thinking about the new coronavirus
4. You fear losing your life to the new coronavirus.
5. When you see news or talk about the new coronavirus on the Internet, you get nervous or anxious
6. You can't sleep because you are worried about the new coronavirus infection.
7. Thinking about new coronavirus infection causes rapid heartbeat and palpitations
8. You fear losing your life due to cigarette smoke.
9. You fear losing your life due to heavy drinking.
10. You fear losing your life due to a car accident.

<Choice>

1. Not applicable at all (0%)
2. Not applicable (25%)
3. Neither (50%)
4. Applicable (75%)
5. Very applicable (100%)

(Q45) Did you obtain information about the new coronavirus infection from any of the following?

1. Family
2. Friends and acquaintances
3. Workplace / school
4. Family doctors and other health care professionals
5. Famous celebrities
6. Specialist
7. Websites of public offices (Ministry of Health, Labor and Welfare, and prefectural and municipal governments)
8. Websites of universities, academic societies, and other research institutions
9. Private video sites (e.g., YouTube)
10. Line
11. Twitter
12. Facebook
13. Instagram
14. Usenet
15. Newspaper
16. Magazine
17. Book
18. Television (news)
19. Television (comprehensive show)
20. Radio

<Choice>

1. Yes

2. No

(Q46) How much do you trust the information sources you selected in the previous question?

1. Family
2. Friends and acquaintances
3. Workplace / School
4. Family doctors and other health care professionals
5. Famous Celebrities
6. Specialist
7. Websites of public offices (Ministry of Health, Labor and Welfare, and prefectural and municipal governments)
8. Websites of universities, academic societies, and other research institutions
9. Private video sites (e.g., YouTube)
10. Line
11. Twitter
12. Facebook
13. Instagram
14. Usenet
15. Newspaper
16. Magazine
17. Book
18. Television (news)
19. Television (comprehensive show)
20. Radio

<Choice>

1. Very much trust it.
2. Trust it.
3. Rather trust it.
4. Rather not trust it.
5. Don't trust it.
6. Don't trust it at all.

(Q47) Do you think you would be able to find or use information related to your illness or health on your own if you needed to? Answer each of the following.

1. You can gather information from a variety of sources, including newspapers, books, television, and the Internet
2. You can pick out the information you want from such information.
3. You can understand and communicate information to others
4. You can determine how reliable the information is
5. You can make informed decisions about plans and actions to improve health

<Choice>
1. I disagree.
2. I don't think so.
3. Neither.
4. I think so.
5. I strongly agree.

(Q48) Have you experienced any of the following events from April 2020 – the present?
1. Felt blocked up and did not want to talk to anyone because of anxiety
2. Could not sleep as well as you usually do.
3. No one contacted you, and you felt isolated.
4. The rules of your life have been disrupted.
5. Someone close to you has passed away.
6. Physically assaulted, such as being hit, kicked, thrown objects at, or locked in
7. Received verbal abuse, sarcasm, long periods of neglect, or other actions that hurt your self-esteem
8. Your deposit or pension was used or taken without your consent (including by family members)
9. There was non-consensual sexual activity.
10. There was anxiety about pregnancy without testing.
11. Hesitated to go out because of people watching
12. Worried that people would accuse me of being infected with the new corona virus.
13. Felt discriminated against in any way related to the new coronavirus infection
14. Felt as if you were being monitored about your behavior regarding infection prevention (e.g., masking, hand washing, social distancing, etc.)
15. Others have warned you about your behavior regarding infection prevention (e.g., masking, hand washing, ensuring social distancing, etc.)
16. Concerned about others' actions related to infection prevention (e.g., masking, hand washing, ensuring social distancing, etc.)
17. Attention was paid to those who did not take infection prevention actions (e.g., not wearing a mask, not washing hands, not ensuring social distancing, etc.)
18. Using the GO TO Travel Campaign (Business)
19. Online meeting to your parents.

<Choice>
1. Yes
2. No

(Q49) In the last 30 days, how often did you experience any of the following?
1. Did you feel oversensitive?
2. Did you feel it was hopeless?
3. Did you feel fidgety and restless?
4. Did you feel depressed and like you didn't feel good about what was happening?

5. Did you feel you had to do to make ends meet somehow?
6. Did you feel you were worthless?
7. Did you feel that you did not have any social interaction?
8. Did you feel left out?
9. Do you ever feel that you were isolated from others?
10. Compared to before the novel coronavirus pandemic (before January 2020), did you feel more isolated from your surroundings?

<Choice>

1. Always
2. Mostly
3. Sometimes
4. Just a little.
5. Not at all.

Question display condition: Display 1, 2, 5, 8, 9, 10 with male or female gender.

Question display condition: (Q3) 1 is 1 or more, and 3 is displayed.

Question display condition: 1 in (Q5) is 1, 5 in (Q5) is 5, and 6 and 7 are displayed.

Question display condition: 2 in (Q3) + 3 in (Q3) is 1 or more, or 4 is displayed if the age is between 30 and 150.

(Q50) Compared to before January 2020, how has each of the following changed in the last month?

1. Your state of mental health
2. Your state of physical health
3. Your relationship with your spouse
4. Your relationship with your child
5. Your relationship with family members other than spouse and children
6. Your relationship with your boss at work
7. Your relationship with your subordinates and colleagues at work
8. Your relationship with your friends
9. Overall work/study performance (performance)
10. Overall housekeeping performance (performance)

<Choice>

1. Much better.
2. Somewhat improved
3. The same
4. Slightly worse.
5. Much worse.
6. I don't know.

Question display condition: Display 1, 2, 5, 8, 9, 10 with male or female gender.

Question display condition: 1 in (Q5) is 1, 2 in (Q5) is 1, 3 in (Q5) is 1, 4 in (Q5) is 1, 5 in (Q5) is 1, and (Q5-1) is 10, 7 is displayed.

Question display condition: 1 in (Q5) is 1, 2 in (Q5) is 1, 3 in (Q5) is 1, 4 in (Q5) is 1, 5 in (Q5) is 1, and 8 is displayed.

Question display condition: 9 is displayed when (Q20) is 1-4.

(Q51) What do you think about the following items? Please indicate your current perception.

1. People in your community are generally reliable
2. People in your community often try to help others
3. The request to refrain from leaving the house based on the declaration of a state of emergency was satisfactory.
4. Government can be trusted.
5. If you or your family member got a coronavirus infection, you would want to keep it a secret.
6. You hesitate to associate with people who got coronavirus infection, even after they have recovered from the disease.
7. You can take a day off from work or school when you catch a cold.
8. You feel that the way you work after April is more suitable for yourself.
9. You may lose your job within 3 months from now.
10. You may lose your place of residence within 3 months from now.

<Choice>

1. I strongly agree.
2. I think so.
3. I don't think so.
4. I strongly disagree.

(Q52) How much do the following items apply to you?

1. You are lively and extroverted.
2. You are dissatisfied with others and prone to getting into trouble.
3. You are solid and hard on yourself.
4. You are prone to worrying and fretting.
5. You like new things and different ideas.
6. You are reserved and quiet.
7. You are a kind person who cares about others.
8. You are sloppy and careless.
9. You are calm, and your mood is stable.
10. You are mediocre and lacking in ideas.

<Choice>

1. I strongly disagree.
2. I don’t think this applies to me.
3. I think I am a bit different.

4. Neither agree nor disagree.
5. I think so a little.
6. I guess so.
7. I strongly agree.

(Q53) During the past month, have you had opportunities to inhale smoke (passive smoking) from cigarettes (excluding heated cigarettes) that people other than yourself smoked? For each place, please choose one that applies. Heated cigarettes include Icos, Glow, and Plumtec.

<Location>

1. Family
2. Workplace
3. School
4. Restaurant (esp. Western-style)
5. Coffee shop
6. Pub/Bar
7. Pachinko parlor
8. Inside the car
9. Road

<Choice>

1. Almost every day
2. A few times a week
3. About once a week
4. About once a month
5. There was none at all.
6. I never went to this place.

(Q54) In the past month, have you had the opportunity to breathe the vapor or mist (aerosol) of a heated cigarette used by someone other than yourself? For each location, please choose one that applies. *Heated cigarettes include Icos, Glow, and Bloomtec.

<Location>

1. Family
2. Workplace
3. School
4. Restaurant (esp. Western-style)
5. Coffee shop
6. Pub/Bar
7. Pachinko parlor
8. Inside the car
9. Road

<Choice>

1. Almost every day
2. A few times a week
3. About once a week
4. About once a month
5. There was none at all.

(Q55) Do you currently drink or use alcohol or drugs? Please answer each of the following.
1. Alcohol (beer, sake, shochu, wine, whiskey, etc.)
2. Sleeping pills/anti-anxiety medications
3. Narcotics such as morphine (prescribed by a physician and used for cancer pain)
4. Narcotics such as morphine (prescribed by a physician and used for non-cancer pain)
5. Narcotics such as morphine (obtained in a manner not prescribed by a physician)
6. Inhalation of organic solvents such as thinner and toluene (except for appropriate use on the job)
7. Dangerous drugs (e.g., law-evading herbs, magic mushrooms)
8. Cannabis (marijuana)
9. Methamphetamine, cocaine, heroin

<Choice>
1. Never used it before.
2. Tried using it more than once but did not use it habitually
3. I used to use it habitually, but I have stopped.
4. Some days, I use it from time to time.
5. I use it almost every day.

(Q56) Do you currently smoke or use tobacco? Please answer each of the following.
1. Cigarettes
2. Hand-rolled cigarettes (roll your cigarettes using a kit or other means to make paper cigarettes)
3. Ploom Tech
4. Ploom Tech + (Plum Tech Plus)
5. Ploom S
6. IQOS
7. Glo
8. Glo sens
9. PULZE
10. E-cigarettes (those containing nicotine)
11. E-cigarettes (without nicotine)
12. E-cigarettes (which may or may not contain nicotine)
13. Cigar
14. Little Cigar
15. Pipe
16. Flue pipe

17. Chewing tobacco
18. Snuffing tobacco
19. Hookah

<Choice>

1. Never smoked (or used) before.
2. Smoked (used) more than once but did not use habitually
3. Used to smoke (use) habitually but have stopped now
4. Sometimes, smoke (use) days.
5. I smoke (and use) it almost every day.

(Q57) How is your current health condition?

<Choice>

1. Very good
2. Good
3. Average
4. Not so good.
5. Not good

(Q58) What was your household's annual income (including taxes) in 2019? Please choose one number that applies. Household income is the total income earned by your home as a whole during 2019 (the first year of 2019). (Including income from work and other income such as remittances from parents, property income such as rent, child allowance, etc.)

1. No household income
2. Less than 0.5 million yen
3. More than 0.5 million yen but less than 1 million yen
4. More than 1 million yen but less than 2 million yen
5. More than 2 million yen but less than 3 million yen
6. More than 3 million yen but less than 4 million yen
7. More than 4 million yen but less than 5 million yen
8. More than 5 million yen but less than 6 million yen
9. More than 6 million yen but less than 7 million yen
10. More than 7 million yen but less than 8 million yen
11. More than 8 million yen but less than 9 million yen
12. More than 9 million yen but less than 10 million yen
13. More than 10 million yen but less than 12 million yen
14. More than 12 million yen but less than 14 million yen
15. More than 14 million yen but less than 16 million yen
16. More than 16 million yen but less than 18 million yen
17. More than 18 million yen but less than 20 million yen
18. More than 20 million yen

19. I don't want to answer.
20. I don't know.

(Q59) Please select all (money) public assistance you have received from April 2020 to the present.
<Choice>

1. Special fixed benefit (0.1 million yen per person)
2. Employment adjustment subsidy for employers (notable exception due to the impact of the new coronavirus infection)
3. Temporary special benefits for households raising children
4. Sustaining benefits (for sole proprietorships and small and medium-sized corporations)
5. Housing security benefits
6. Public assistance
7. Unemployment allowance
8. Disability allowance
9. Nursing allowance
10. Child-care allowance
11. Other public assistance
12. Received none of them.

(Q60) Do you think you are happy? Please choose one number.
<Choice>

1. 10 points Very happy
2. 9 points
3. 8 points
4. 7 points
5. 6 points
6. 5 points
7. 4 points
8. 3 points
9. 2 points
10. 1 point Not happy

(Q61) Do you currently have any chronic illnesses?

1. High blood pressure
2. Diabetes mellitus
3. Asthma
4. Bronchitis, pneumonia
5. Atopic dermatitis
6. Periodontal disease
7. Dental caries (tooth decay)

8. Otitis media
9. Angina pectoris
10. Myocardial infarction
11. Stroke (cerebral infarction or hemorrhage)
12. COPD (chronic obstructive pulmonary disease)
13. Cancer and malignancies
14. Chronic pain such as back pain and headache (lasting longer than 3 months)
15. Depression
16. Mental illness other than depression

<Choice>

1. I have never had this illness.
2. Not in the present, but in the past.
3. Present (commuting to hospital)
4. Present (not commuting to hospital)

Supplementary 2: The definition of each region.

Hokkaido area: Hokkaido
Tohoku area: Aomori, Iwate, Miyagi, Akita, Yamagata, Fukushima
Kita-Kanto area: Ibaragi, Tochigi, Gunma
Tokyo area: Saitama, Tokyo, Chiba, Kanagawa
Chubu-Hokuriku area: Niigata, Toyama, Ishikawa, Fukui, Yamanashi, Nagano, Shizuoka
Chukyo area: Gifu, Aichi, Mie
Osaka area: Kyoto, Osaka, Hyogo
Keihan area: Shiga, Nara, Wakayama
Chugoku area: Tottori, Shimane, Okayama, Hiroshima, Yamaguchi
Shikoku area: Tokushima, Kagawa, Ehime, Kochi
Kyusyu-Okinawa area: Fukuoka, Saga, Nagasaki, Kumamoto, Oita, Miyazaki, Kagoshima, Okinawa

Supplementary figure S1: Distribution of subjective well-being of pregnant women in 2020 and 2021

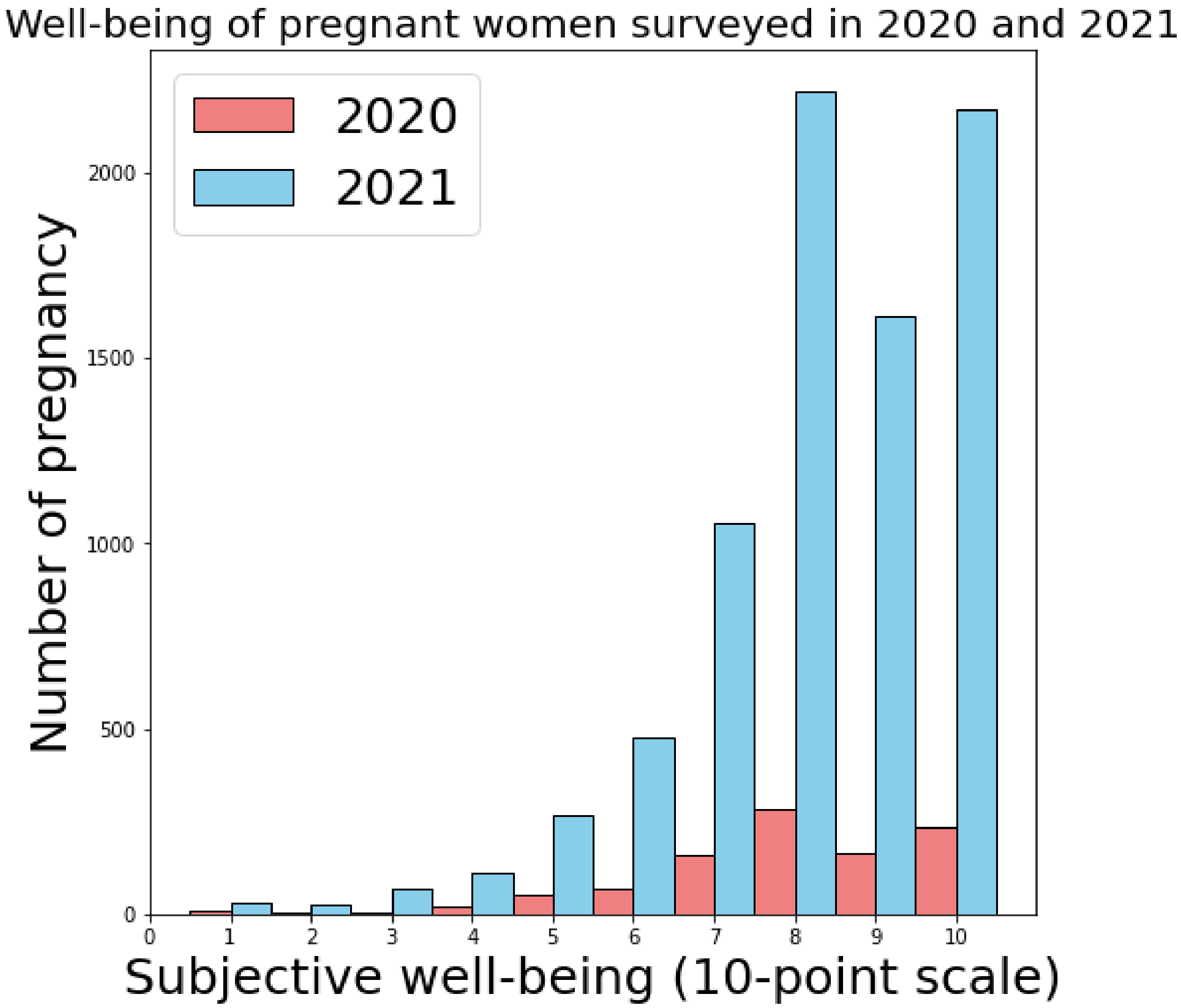

Supplementary figure S2: Receiver operating characteristic (ROC) curves for models trained and tested using 2020 data

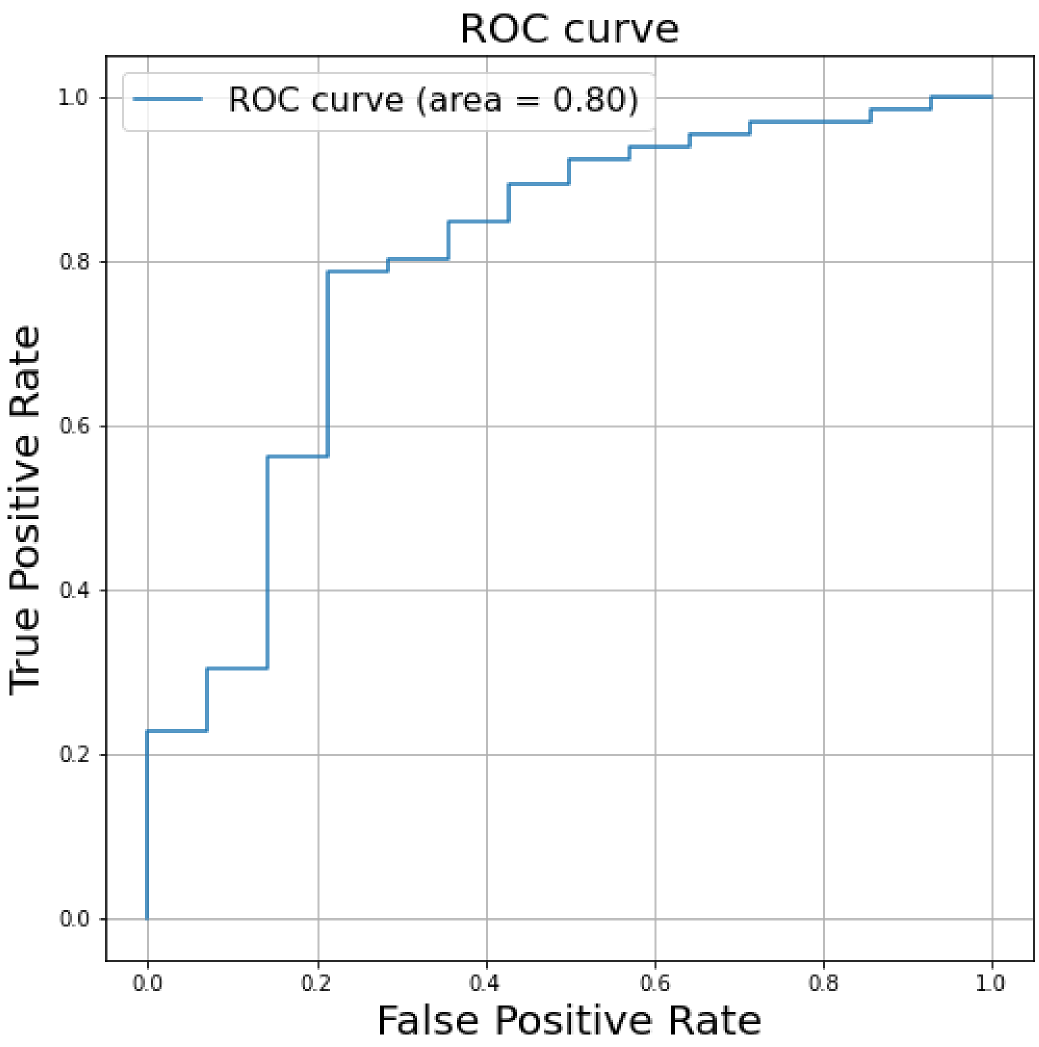

Supplement: Supplementary file 1 — Supplementary Information. [file 41598_2023_44410_MOESM1_ESM.pdf]
